# Prognostic biomarkers of biliary atresia—are we there yet?

**DOI:** 10.1038/s41390-025-04034-9

**Published:** 2025-07-22

**Authors:** Sunitha Vimalesvaran, Barath Jagadisan, Anil Dhawan

**Affiliations:** Department of Paediatric Gastroenterology, Hepatology and Nutrition, King’s College Hospital, London, UK

## Abstract

Biliary atresia (BA) is a progressive cholangiopathy and the leading cause of pediatric liver transplantation. While its etiology remains unclear, factors such as developmental anomalies, viral infections, and immune dysregulation have been implicated. Early Kasai portoenterostomy (KPE) is the standard surgical intervention to restore bile flow, with serum bilirubin normalization serving as a key success indicator. Even after successful KPE, progressive fibrosis remains a major challenge. Recent research has focused on identifying biomarkers for BA prognosis, ranging from indicators of cholangitis and portal hypertension to predictors of jaundice clearance and native liver survival. The study by Taylor et al. explores early immune signatures predicting post-KPE biliary drainage in BA. Their findings revealed that increased monocyte-like macrophages (MLM) and elevated granulocyte-macrophage colony-stimulating factor (GM-CSF) levels correlated with improved bile flow post-KPE. The authors suggest that GM-CSF-driven macrophage polarization may enhance an anti-inflammatory response, potentially influencing fibrosis progression and long-term liver function. While these findings provide valuable insight into immune-mediated mechanisms in BA, we show there remains ambiguity around the clinical utility of biomarkers in BA. Studies are still needed to validate these prognostic biomarkers and improve risk stratification in these patients.

Biliary atresia (BA), a fibro-inflammatory cholangiopathy of the extra- and intrahepatic bile ducts, is the leading cause of biliary cirrhosis and liver transplantation in the pediatric population. Whilst the exact etiology remains unknown, studies have suggested an error in the developmental process, viral infections, or a dysfunctional immune response.^1^ Early Kasai portoenterostomy (KPE) surgery is the accepted treatment in an attempt to re-establish bile flow. Normalization of serum bilirubin levels to <2 mg/dl is regarded as the success parameter for KPE. Failure to establish adequate bile flow leads to rapid progression of liver dysfunction, necessitating liver transplantation, usually by 2 years of age. However, despite a successful KPE, there is often an unpredictable progression of fibrosis in children who are jaundice-free, leading to later onset of cirrhosis, portal hypertension, and synthetic failure of the liver necessitating liver transplantation. Other indications for liver transplantation include recurrent cholangitis, hepatopulmonary syndrome, or other rare complications of cirrhosis.^2–4^

Biomarkers have been studied in biliary atresia (i) for diagnostic purposes to differentiate biliary atresia from other causes of cholestasis, (ii) to define or corroborate a diagnosis of cholangitis after KPE, (iii) to estimate the degree of portal hypertension and the need for surveillance endoscopy and (iv) to predict jaundice clearance after KPE and native liver survival. Predicting native liver survival remains an area of active research since prognostic markers described in the past have not stood the test of time.^5,6^ Prognostication not only helps in counseling parents and patients but also defines patient sub-populations that are homogenous in their outcomes, which in turn could be the target for focused monitoring and interventions, including experimental therapies. The prognosis in BA is probably a function of the instantaneous degree of architectural distortion in the liver, the degree of effective biliary drainage, ongoing inflammation in the liver, and the impact of recurrent cholangitis. The degree of bile drainage in itself may be influenced by the architectural distortion and also the surgical technique in the early phase. Hence, biomarkers at diagnosis may not be entirely reflective of late outcomes. They may impact early outcomes after surgery, although confounded by variations in surgical techniques and expertise. This would also probably imply that prognostication in BA would have to be dynamic and based on composite biomarkers and clinical events addressing the various factors discussed above. There is a hope that in the future, biomarkers may indicate the activity of specific mechanistic drivers like inflammation and fibrosis that can be specifically addressed when such targeted therapies are available.

Prognostic biomarkers in the past initially addressed the efficacy of bile drainage in the form of serum bilirubin levels. More recently, serum bile acid levels have been shown to be useful prognostic markers.^7^ Serum matrix metallopeptidase-7 (MMP-7) has also been extensively investigated in the pathogenesis of BA as well as in its predictive value post-KPE. Several studies have shown that a high serum concentration of preoperative MMP-7 correlates positively with the severity of post-KPE liver fibrosis.^8–11^ MMP-7 protein was also found to be significantly elevated in BA patients with persistent cholestasis and liver fibrosis.^12–14^ Serum hyaluronic acid (HA) concentration is increased in patients with chronic liver disease due to the impaired uptake of HA. This has been postulated to be a sensitive marker of liver impairment, with a few studies suggesting that a higher post-KPE serum HA is associated with the severity of liver fibrosis.^15–18^ Aspartate aminotransferase to platelet ratio index (APRi) has been shown in a number of case-control studies to be a sensitive non-invasive biomarker for predicting post-KPE liver fibrosis and cirrhosis.^19^ In one meta-analysis, based on different cut off values, the summary sensitivity and specificity of 2 studies included was 61% and 80%, respectively.^20,21^ The studies showed that APRi had a high diagnostic accuracy for predicting post-KPE significant fibrosis and cirrhosis. However, no association between pre-operative APRi and native survival has been observed thus far. Of late, biomarkers of inflammation have attracted equal attention as predictors of the outcome of survival with native liver.

Wang et al. profiled mononuclear immune cells from liver biopsies and blood samples of infants with BA and compared that to profiles of non-BA cholestatic and non-cholestatic babies as control subjects.^22^ They demonstrated that liver damage in BA correlated with both immune-mediated destruction and reduced immune restoration. This was linked to higher cytotoxic-type immunity (CD8Trm and Th1) but decreased Th17-type immunity (cTh17, MAIT17, and γδT17). This study also showed that the concentrations of self-reactive natural IgM antibodies were decreased (important protective role against common infections and autoimmune diseases) and a higher concentration of high-affinity pathogenic IgG antibodies. Evidence suggests that immune pathogenic mechanisms play an important role in triggering BA but is also key in the postoperative period, leading to ongoing damage and progression of liver disease.^23,24^

The study by Taylor et al. in this issue of Pediatric Research studied a number of early immune signatures associated with biliary drainage after KPE.^25^ The study is based on the data from the NIDDK-supported Childhood Liver Disease Research Network. The authors analyzed 36 cytokines and hepatic immune cell subsets in a cohort of 38 infants with BA (20 with successful bile flow after KPE; 18 without) and 17 non-BA cholestatic infants at diagnosis. They showed that monocyte like macrophages (MLM) and serum levels of granulocyte-macrophage colony stimulating factor (GM-CSF) were increased in BA participants with good biliary drainage (*p* = 0.004 and *p* < 0.001 respectively). The data suggests that the presence of hepatic MLM and serum GM-CSF cytokine levels early in the disease course can predict favorable post-KPE biliary drainage. In addition, GM-CSF polarization of MLM at the time of diagnosis may promote an anti-inflammatory immune response that could also contribute to biliary flow post-KPE.

The colony stimulating factor (CSF) superfamily, namely macrophage (M-CSF), granulocyte (G-CSF), and GM-CSF were originally defined as growth factors (GF) capable of promoting myeloid colony formation from bone marrow precursors in vitro. However, unlike M-CSF and G-CSF, GM-CSF is not typically required for steady-state myelopoiesis.^26^ There is growing evidence, however, that an increased concentration of GM-CSF is found at sites of tissue inflammation. This has been found in patients with late-phase cutaneous reactions, rheumatoid arthritis, and cerebrospinal fluid in patients with multiple sclerosis.^27–29^ These observations are supported by data from several preclinical models, suggesting importance of GM-CSF in promoting inflammation.^30^ The central role of GM-CSF in inflammation as a conduit between tissue invading lymphocytes and myeloid cells has justified the development of clinical trials evaluating GM-CSF blockade.^31^ Whilst primarily pro-inflammatory, GM-CSF also has potential anti-inflammatory and tissue repair roles. GM-CSF has been shown to polarize macrophages towards a reparative phenotype (M2-like macrophages), promoting tissue remodeling and resolution of fibrosis.^32^ It has also been shown to mobilize bone marrow-derived stem and progenitor cells, which may contribute to liver regeneration.^33^ The role of GM-CSF as an immune-modulatory cytokine in BA remains unclear.

In cholestastic hepatic disorders, various GF have been shown to be elevated during fibrotic processes. This was hypothesized to be due to the effect of GF in the regulation of liver stem cell differentiation.^34^ Madadi-Sanjani showed high levels of GF including GM-CSF at the time of KPE but no correlation to the outcome of the 49 infants included could be established. They concluded that local or systemic GF levels at time of KPE remains unsuitable as a predictive tool for the disease course in BA.^35^

The present study by Taylor et al. however, distinguishes itself from the published literature by demonstrating the interaction of GM-CSF with a larger range of cell types including monocytes/ macrophages. They suggest that the increased levels of hepatic MLM in BA patients with good outcome after KPE may be secondary to GM-CSF-driven macrophage polarization and is potentially beneficial in BA.

Nevertheless, the evidence remains scarce with ambiguity of its utility in the broader context. While biomarkers like GM-CSF show promise, their application in clinical practice is hindered by limitations in sample sizes, heterogeneity in disease phenotypes, and variability in study methodologies. In disorders such as biliary atresia where there is an element of ‘centre- effect’ in outcomes as a result of surgical expertise, multicentric studies that include outcome data from low volume studies tend to create a level of heterogeneity. Validation of prognostic biomarkers are best done either at single or multiple centers that handle large volumes of cases in order to refine their prognostic accuracy. Moreover, a deeper understanding of the interplay between GF, immune cell subsets, and fibrotic pathways in BA may pave the way for novel therapeutic interventions aimed at modulating immune responses and improving native liver survival.

BA remains a complex and enigmatic disease, challenging clinicians and researchers alike. Despite advancements in the field, the prognosis for many children is guarded, with the majority progressing to liver fibrosis and eventual transplantation. Research into the immunopathogenesis of BA has highlighted the critical role of immune-mediated mechanisms, which not only contribute to disease onset but also perpetuate progression post-surgery. Recent findings, including those by Taylor et al., underscore the potential of immune biomarkers such as GM-CSF and hepatic MLM in predicting post-KPE outcomes. These insights open avenues for stratifying patients early in their disease course and tailoring management strategies. The integration of immune-based biomarkers into the clinical management of BA represents an exciting frontier. Collaborative efforts across laboratory research and clinical domains will be essential to translate these discoveries into meaningful improvements in outcomes for children affected by this devastating disease.
